# Simvastatin Reduces Protection and Intestinal T Cell Responses Induced by a Norovirus P Particle Vaccine in Gnotobiotic Pigs

**DOI:** 10.3390/pathogens10070829

**Published:** 2021-07-01

**Authors:** Jacob Kocher, Tammy Bui Castellucci, Ke Wen, Guohua Li, Xingdong Yang, Shaohua Lei, Xi Jiang, Lijuan Yuan

**Affiliations:** 1 Center for Emerging, Zoonotic, and Arthropod-Borne Pathogens, Department of Biomedical Sciences and Pathobiology, Virginia-Maryland College of Veterinary Medicine, Virginia Polytechnic Institute and State University, Blacksburg, VA 24061, USA; jacobk09@vt.edu (J.K.); hibiscus@vt.edu (T.B.C.); wenke316@vt.edu (K.W.); lguohua@vt.edu (G.L.); xingy86@vt.edu (X.Y.); lsh2013@vt.edu (S.L.); 2 Division of Infectious Diseases, Cincinnati Children’s Hospital Medical Center, Cincinnati, OH 45229, USA; Jason.Jiang@CCHMC.org; 3 Department of Pediatrics, University of Cincinnati College of Medicine, Cincinnati, OH 45229, USA

**Keywords:** simvastatin, human norovirus, P particle vaccine, gnotobiotic pig, T cells, protective efficacy

## Abstract

Noroviruses (NoVs) are a leading cause of acute gastroenteritis worldwide. P particles are a potential vaccine candidate against NoV. Simvastatin is a cholesterol-reducing drug that is known to increase NoV infectivity. In this study, we examined simvastatin’s effects on P particle-induced protective efficacy and T-cell immunogenicity using the gnotobiotic pig model of human NoV infection and diarrhea. Pigs were intranasally inoculated with three doses (100 µg/dose) of GII.4/VA387-derived P particles together with monophosphoryl lipid A and chitosan adjuvants. Simvastatin-fed pigs received 8 mg/day orally for 11 days prior to challenge. A subset of pigs was orally challenged with 10 ID_50_ of a NoV GII.4/2006b variant at post-inoculation day (PID) 28 and monitored for 7 days post-challenge. Intestinal and systemic T cell responses were determined pre- and postchallenge. Simvastatin abolished the P particle’s protection and significantly increased diarrhea severity after NoV infection. Simvastatin decreased proliferation of virus-specific and non-specific CD8 T cells in duodenum and virus-specific CD4 and CD8 T cells in spleen and significantly reduced numbers of intestinal mononuclear cells in vaccinated pigs. Furthermore, simvastatin significantly decreased numbers of duodenal CD4+IFN-γ+, CD8+IFN-γ+ and regulatory T cells and total duodenal activated CD4+ and CD8+ T cells in vaccinated pigs pre-challenge at PID 28. Following challenge, simvastatin prevented the IFN-γ+ T cell response in spleen of vaccinated pigs. These results indicate that simvastatin abolished P particle vaccine-induced partial protection through, at least in part, impairing T cell immunity. The findings have specific implications for the development of preventive and therapeutic strategies against NoV gastroenteritis, especially for the elderly population who takes statin-type drugs.

## 1. Introduction

Noroviruses (NoVs) are a member of the *Caliciviridae* family and a leading cause of acute, non-bacterial gastroenteritis [1]. NoVs account for approximately 20% of all cases of gastroenteritis worldwide with GII.4 causing 60–90% of all outbreaks, but new GII variants are increasingly become predominate [2,3,4]. NoVs cause an estimated 20 million illnesses and $10.6 billion in clinical and economic losses in the United States each year [5]. NoVs are easily transmitted in semi-closed communities, such as senior care homes and cruise ships [6,7,8]. NoV infections are known to cause more severe disease in the elderly population [9]. In the United States, NoV is the second only to *Clostridium difficile* as a cause of death from gastroenteritis, responsible for nearly 800 deaths annually in patients older than 65 years of age [10]. 

NoV vaccines are the most effective approach to prevent NoV disease and the development of NoV vaccine is progressing. Phase I and II human clinical trials of virus-like particle (VLP) vaccines in healthy adults have shown proof-of-principle that the vaccines are immunogenic and can reduce illness and infection [11,12]. P particles are another novel vaccine candidate derived from expression of the VP1 protruding (P) domain in a prokaryotic expression vector [13,14,15]. Previously, we reported that an intranasal three-dose 100 µg P particle regimen provided a similar protection rate as VLPs against diarrhea following cross-variant, homotypic NoV challenge in gnotobiotic (Gn) pigs [16]. Both regimens provided similar protection as the intranasal two-dose 100 µg Norwalk-derived VLPs in humans [17]. Additionally, P particles primed for increased activated CD4+ T cells, duodenal CD8+IFN-γ+ T cells, CD25—FoxP3+ regulatory T cells (Tregs) in PBL and CD25—FoxP3+TGF-β+ Tregs in spleen compared to VLPs following NoV challenge in Gn pigs [16]. 

Simvastatin is a cholesterol-reducing drug that inhibits HMG-CoA reductase, an enzyme in the cholesterol biosynthesis pathway, resulting in reduction of low-density lipoprotein (LDL) cholesterol levels [18]. Forty milligrams of simvastatin decreases LDL cholesterol and the risk of cardiovascular events by 23% over 5 years [19] with similar effects witnessed in low-risk populations [20]. Based on the report by the National Center for Health Statistics, 50% of men and 36% of women who are 65–74 years old took statin type drugs in 2010. In 2013, the American Heart Association and American College of Cardiology released new guidelines which expand the recommendation for the use of simvastatin in the prevention of heart diseases even to people without high LDL levels. 

Simvastatin has been shown to reduce the severity of several other diseases, including rheumatoid arthritis [21,22,23], multiple sclerosis [24] and periodontitis [25,26]. These pleiotropic effects can be explained by simvastatin’s role in the down-regulation of IFN-induced MHC II expression by inhibition of the *CIITA* gene [27,28]. This down-regulation of induced MHC II has many downstream effects, including reduced NK cell cytotoxicity [29,30], reduced in vitro T cell proliferation [30], reduced production of IL-2 and IFN-γ [31], reduced CD4/CD8 and Th1/Th2 ratios [22], increased Tregs [32,33] and impaired lymphocyte homing to secondary lymphoid organs [34].

Cholesterol pathways have been shown to play a role in murine NoV and bovine NoV VLP cellular entry [35,36,37] and in Norwalk replication [38]. Norwalk replication was directly associated with increased expression of LDL receptor (LDLR) mRNA in cells bearing the Norwalk replicon [38]. Simvastatin has been shown to increase the expression of LDLR [18] and the production of Norwalk proteins and RNA in replicon-bearing cells [38]. Additionally, 25-hydroxycholesterol has been shown to reduce murine NoV replication in RAW264.7. cells [39]. During Hepatitis E virus infection, simvastatin treatment led to a significantly increased viral release in vitro and elevated viral loads in the patient sera [40].

The roles of simvastatin on NoV infection and disease have also been investigated in vivo. Gn pigs treated with simvastatin had earlier onset and longer duration of fecal NoV shedding, as well as increased viral titers compared to pigs not fed simvastatin [41]. Oral inoculation of IFN-α reduced the effects of simvastatin on NoV infectivity, indicating simvastatin down-regulates innate immunity [41]. Previously, our study showed that simvastatin feeding increased the susceptibility of Gn pigs to infection by a NoV GII.4 variant, increased incidence of diarrhea compared to non-simvastatin fed Gn pigs and reduced the ID_50_ of the GII.4 variant in Gn pigs [42]. 

In this study, we used the well-established Gn pig model to evaluate the effects of simvastatin on the protective efficacy of the P particles vaccine following cross-variant GII.4 NoV challenge. Since simvastatin’s immunomodulatory effects have been previously shown to mainly impact T cell responses [22,43,44], we examined simvastatin’s effects on the T cell profile induced by the P particles in the intestinal and systemic lymphoid tissues in Gn pigs. Simvastatin is primarily consumed by the elderly and aging, one of the target populations for NoV vaccines [45], but its effects on NoV vaccine-induced immunity have not been investigated. 

## 2. Results

### 2.1. Simvastatin Feeding Significantly Reduced Serum Cholesterol in Gn Pigs 

Serum cholesterol was monitored before and after simvastatin feeding to verify its effects in Gn pigs. Simvastatin feeding significantly reduced serum cholesterol after 11 days of feeding. Mean serum cholesterol levels decreased from 133 mg/dL at post-inoculation day (PID) 17 to 72 mg/dL at PID 27 for a 44% reduction (*p* < 0.0001). Mean serum cholesterol levels in age-matched non-simvastatin fed pigs were 110 mg/dL at PID 17 and 99 mg/dL at PID 27 for an 8% reduction (*p* > 0.05).

### 2.2. Protective Efficacy Conferred by the P Particle Vaccine in Simvastatin-Fed Pigs

The protective efficacy of P particle vaccines against NoV diarrhea and shedding following GII.4/2006b NoV challenge was evaluated in simvastatin-fed pigs. Diarrhea and fecal NoV shedding were monitored from PCD 1 to PCD 7 (Table 1). All P+S+ and CS+ pigs had similar occurrence of diarrhea (100% and 83%). P+S+ and CS+ pigs had significantly higher diarrhea AUCs compared to P+S− pigs (8.8 vs. 5.4). Simvastatin did not have an effect on the occurrence of shedding; however, it reduced the fold-reduction of AUC in vaccinated pigs from the corresponding controls (P+S− 3.1-fold vs. P+S+ 2.3-fold). Additionally, the P particle vaccine significantly shortened the duration of virus shedding (4.2 days vs. 1.7 days) and reduced the mean AUC (2.3-fold) of virus shedding compared to control pigs following NoV challenge (Table 1). The protective effects conferred by the same P particle vaccine against the same GII.4/2006b NoV challenge in non-simvastatin-fed pigs were presented previously [16]. 

### 2.3. Simvastatin Decreases Frequencies of Proliferating Intestinal CD8+ T Cells after Infection

**Figure 1:**
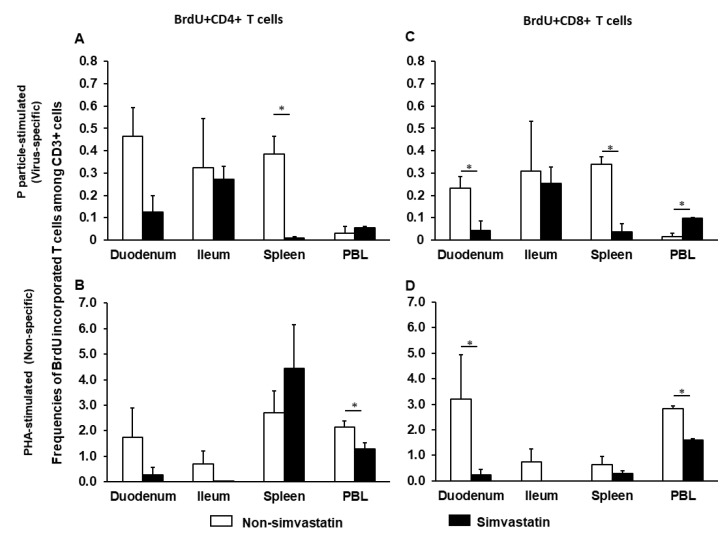
Virus-specific (**A**,**C**) and non-specific (**B**,**D**) proliferating T cells in the presence or absence of simvastatin. MNCs were stimulated with P particles or PHA and cultured with BrdU. Mean frequencies of CD3+CD4+BrdU+ and CD3+CD8+BrdU+ T cells in intestinal (duodenum, ileum) and systemic (spleen, PBL) tissues were analyzed by flow cytometry (Supplementary Figure S1). Note the difference in the Y axis scale between the upper (virus-specific) and lower (non-specific) panels. An asterisk above the bars indicates a significant difference between groups for the same cell type and tissue (*p* < 0.05 by Kruskal-Wallis rank-sum test).

To determine how simvastatin affects proliferating T cells, MNCs were isolated from NoV- vaccinated pigs postchallenge at PCD 7. MNCs were stimulated with P particles (virus-specific) or phytohemagglutinin (PHA) (non-specific), with or without simvastatin and cultured in the presence of BrdU. The frequencies of virus-specific and non-specific proliferating T cells are shown in Figure 1. Simvastatin treatment significantly reduced proliferation of virus-specific CD4+ and CD8+ T cells isolated from the spleen and CD8+ T cells isolated from the duodenum (Figure 1A,C). Curiously, simvastatin significantly increased virus-specific proliferating CD8+ T cells isolated from blood. However, simvastatin significantly reduced the non-specific proliferating CD4+ and CD8+ T cells in PBL and CD8+ T cells in duodenum (Figure 1B,D). Taken together, these results indicate that simvastatin impacts the proliferation of both NoV-specific and non-specific T cells.

### 2.4. Simvastatin Feeding Decreased Total MNCs Isolated from Duodenum Pre-Challenge and PBL Post-Challenge

**Figure 2:**
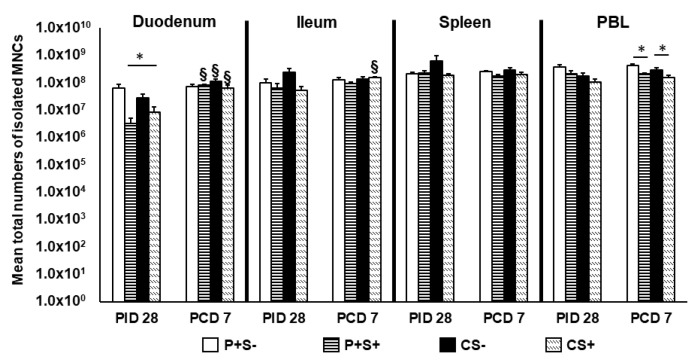
Total numbers of MNCs in intestinal and systemic lymphoid tissues of Gn pigs vaccinated with P particles with or without simvastatin feeding pre- and post-challenge. Total MNC numbers were calculated based on the concentration and total volume of MNCs isolated from the tissues. Total MNC numbers plus standard errors of the means (n = 6 to 10) prechallenge and postchallenge in intestinal (duodenum, ileum) and systemic (spleen, PBL) tissues are presented. An asterisk * above the bars indicates a significant difference among groups for the same cell type and tissue at the same time point; a section sign § indicates that the numbers increased significantly following challenge in the same group (*p* < 0.05 by Kruskal-Wallis rank-sum test). P particles without simvastatin (P+S−), P particles with simvastatin (P+S+), Control without simvastatin (CS−), Control with simvastatin (CS+).

To evaluate how simvastatin feeding affected the development of the neonatal immune system, we calculated the total numbers of MNCs isolated from each tissue pre- and post-challenge (Figure 2). There are four treatment groups designated as (1) P particles without simvastatin (P+S−), (2) P particles with simvastatin (P+S+), (3) Control without simvastatin (CS−) and (4) Control with simvastatin (CS+). The effects of simvastatin in control pigs or vaccinated pigs were revealed by comparisons between CS+ and CS− groups or between P+S+ and P+S− groups, respectively. To assess the immunogenicity of P particles in the presence of simvastatin, the simvastatin-fed vaccinated pigs were compared to simvastatin-fed control pigs (P+S+ vs. CS+). 

At PID 28, simvastatin feeding significantly reduced the total number of duodenal MNCs in vaccinated pigs (P+S− vs. P+S+) and also reduced the numbers in control pigs (CS− vs. CS+). Interestingly, CS+ pigs had significantly higher numbers of MNCs compared to P+S+ pigs in the duodenum. At PCD 7, simvastatin-fed pigs had significantly lower numbers of MNCs in PBL compared to non-simvastatin-fed pigs with or without vaccination. Total MNCs significantly increased in the duodenum of all groups post-challenge except P+S− pigs. Total MNCs also increased significantly in ileum of CS+ pigs post-challenge (Figure 2).

### 2.5. Simvastatin Decreased Total Number and Frequency of CTLs in Duodenum of Vaccinated Pigs Pre-Challenge but Increased Th in PBL Post-Challenge

**Figure 3:**
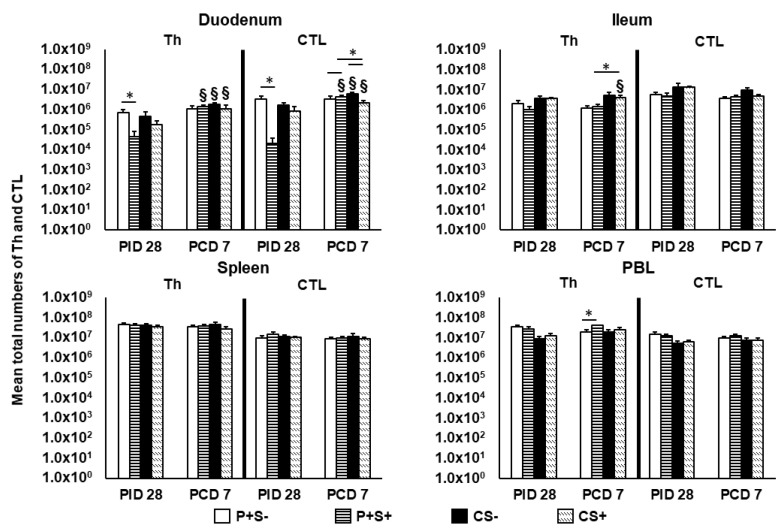
Total numbers of Th (CD3+CD4+) and CTL (CD3+CD8+) in Gn pigs vaccinated with P particles with or without simvastatin feeding pre- and postchallenge. MNCs were gated as previously described [16] following in vitro stimulation (Supplementary Figure S2). Total numbers plus standard errors of the mean (n = 6 to 10) of CD3+CD4+ Th and CD3+CD8+ CTL in intestinal (duodenum, ileum) and systemic (spleen, PBL) tissues pre- and postchallenge are presented. See Figure 2 legend for statistical analysis and an explanation of the symbols indicating statistical significance.

**Figure 4:**
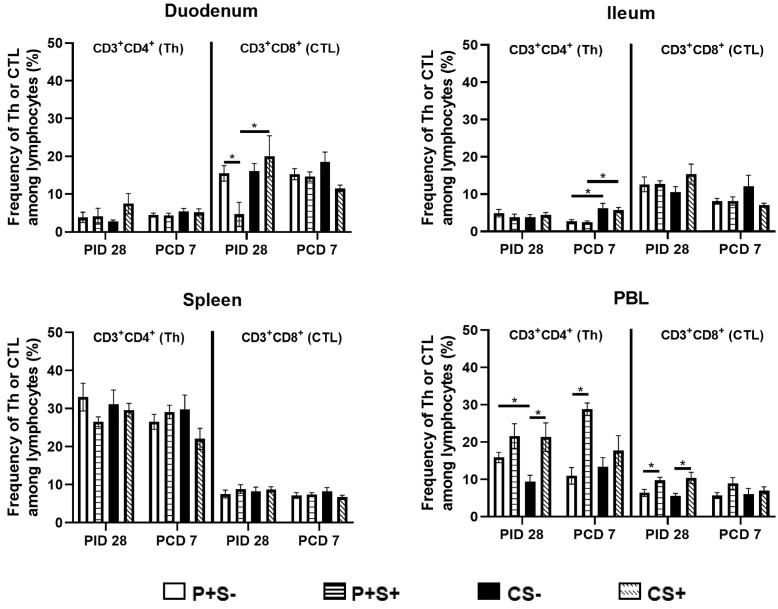
Frequencies of Th (CD3+CD4+) and CTL (CD3+CD8+) in Gn pigs vaccinated with P particles with or without simvastatin feeding pre- and post-challenge. MNCs were gated as previously described [16] following in vitro stimulation (Supplementary Figure S2). Data presented are mean frequencies +/− SEM (n = 6 to 10) of Th or CTL among total lymphocytes in intestinal (duodenum, ileum) and systemic (spleen, PBL) tissue at PID 28 or PCD 7. An asterisk indicates statistical significance among groups for the same cell type at the same time point (*p* ≤ 0.05, Kruskal-Wallis rank-sum test).

To understand the effects of simvastatin on P particle vaccine-induced T cell responses, we evaluated total Th, CTLs, activated non-regulatory CD25+FoxP3— T cells, IFN-γ producing effector/memory T cells and CD4+CD25—FoxP3+ and CD4+CD25+FoxP3+ Tregs in intestinal (duodenum, ileum) and systemic (spleen, blood) lymphoid tissues. The total numbers and frequencies of Th and CTLs were compared among P particle-vaccinated or control pigs with or without simvastatin pre- and post-challenge (Figure 3 and Figure 4). 

At PID 28, P+S+ pigs had significantly lower numbers of Th and CTLs in duodenum compared to P+S− pigs. Following NoV challenge, duodenal Th and CTLs increased significantly from PID 28 to PCD 7 in all groups except P+S− pigs. In duodenum, CS+ pigs had significantly lower numbers of CTLs compared to CS− pigs, but P+S+ pigs had significantly higher numbers of CTLs compared to P+S− and CS+ pigs. In ileum, CS+ pigs had significant increases in Th compared to prechallenge and significantly higher Th compared to P+S+ pigs. In PBL, P+S+ pigs had significantly higher Th numbers compared to P+S− pigs postchallenge (Figure 3). There were no significant differences in the numbers of splenic Th and CTLs among the treatment groups at either timepoints.

When comparing the frequencies of Th and CTLs among P particle-vaccinated or control pigs with or without simvastatin pre- and post-challenge (Figure 4), the overall trends are similar to comparing the total numbers of Th and CTL in each tissue (Figure 3). There are several statistically significant differences observed only in either the number or the frequency data. The most noteworthy difference is that in blood, the frequencies (not the total numbers) of Th and CTL in the simvastatin-fed pigs (P+S+ and CS+) were significantly higher than the non-simvastatin-fed, vaccinated and control pigs (P+S− and CS−) at PID 28 (Figure 4). 

### 2.6. Simvastatin Reduced Numbers and/or Frequencies of Activated CD4+ and CD8+ T Cells in the Intestinal Tissues and Blood Pre-Challenge and CD8+ T Cells in Spleen and Blood Post-Challenge

**Figure 5:**
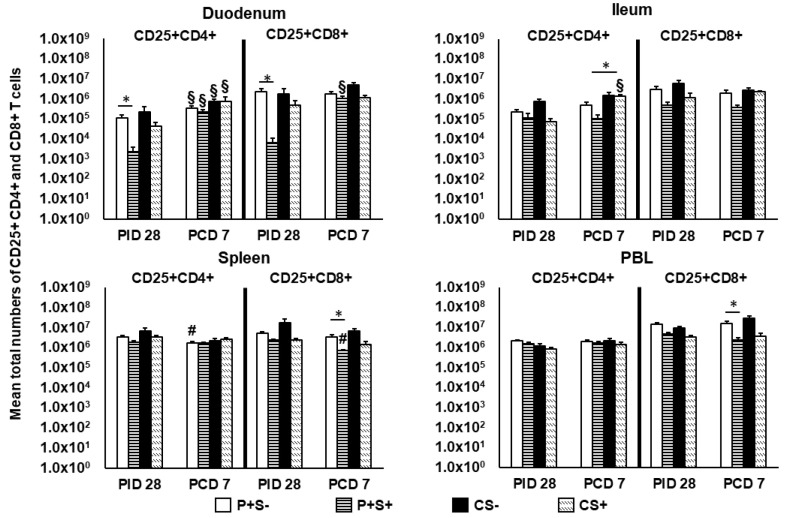
Total numbers of CD25+ expressing CD4+ and CD8+ T cells in Gn pigs vaccinated with P particles with or without simvastatin feeding pre- and postchallenge. MNCs were gated as previously described [16] and analyzed freshly on the day of cell isolation by flow cytometry (Figure S3). Total numbers plus standard errors of the means (n = 6 to 10) of CD4+CD25+FoxP3− and CD8+CD25+FoxP3− activated T cells prechallenge and postchallenge in intestinal (duodenum, ileum) and systemic (spleen, PBL) tissues are presented. See Figure 2 legend for statistical analysis and an explanation of the symbols indicating statistical significance. A number sign # indicates that the cell numbers decreased significantly following challenge in the same group (*p* < 0.05 by Kruskal-Wallis rank-sum test).

**Figure 6:**
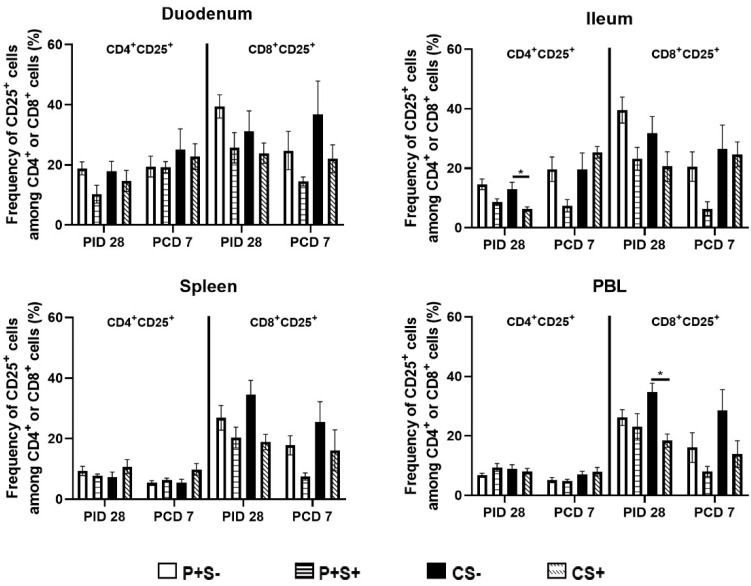
Frequencies of activated CD4+ or CD8+ T cells in Gn pigs vaccinated with P particles with or without simvastatin feeding pre- and post-challenge. MNCs were stained freshly and gated as previously described [16] (Supplementary Figure S3). Data presented are mean frequencies +/− SEM (n = 6 to 10) of FoxP3−CD25+ cells among CD4+ or CD8+ cells in intestinal (duodenum, ileum) and systemic (spleen, PBL) tissue at PID 28 or PCD 7. An asterisk indicates statistical significance among groups for the same cell type at the same time point (*p* ≤ 0.05, Kruskal-Wallis rank-sum test).

To evaluate the effect of simvastatin on T cell activation, activated non-regulatory T cells pre- and post-challenge were enumerated. MNCs were stained freshly on the day of isolation and gated for FoxP3−CD25+CD4+ or FoxP3−CD25+CD8+ T cells. The mean numbers and frequencies of activated CD4+ and CD8+ T cells are shown (Figure 5 and Figure 6, respectively). Pre-challenge, P+S+ pigs had significantly lower numbers of activated CD4+ and CD8+ T cells in duodenum compared to P+S− pigs. Following challenge, the activated CD4+ T cells in duodenum increased significantly from pre-challenge in all groups. P+S+ pigs had significant increases in duodenal activated CD8+ T cells but significant decreases in splenic activated CD8+ T cells. In ileum, CS+ pigs had significant increases in activated CD4+ T cell numbers compared to pre-challenge. Similar to the trend for Th cells, CS+ pigs had significantly higher numbers of activated CD4+ T cells compared to P+S+ pigs in ileum. In spleen and PBL, P+S+ pigs had significantly lower numbers of activated CD8+ T cells compared to P+S− pigs (Figure 5). 

When comparing the frequencies of activated CD4 and CD8 T cells among P particle-vaccinated or control pigs with or without simvastatin pre- and post-challenge (Figure 6), the overall trends are similar to comparing the numbers of activated CD4 and CD8 in most tissues (Figure 5), but the statistically significant differences in the number data were not observed in the frequency data. Two distinct significant differences were detected from the frequency data only. Frequencies of activated CD4+ T cells in ileum and activated CD8+ T cells in blood of simvastatin-fed control pigs (CS+) were significantly lower than the non- simvastatin-fed control pigs (CS−) at PID 28 (Figure 6).

### 2.7. Simvastatin Feeding Reduced Numbers of CD8+IFN-γ+ T Cells in Duodenum and PBL at PID 28

**Figure 7:**
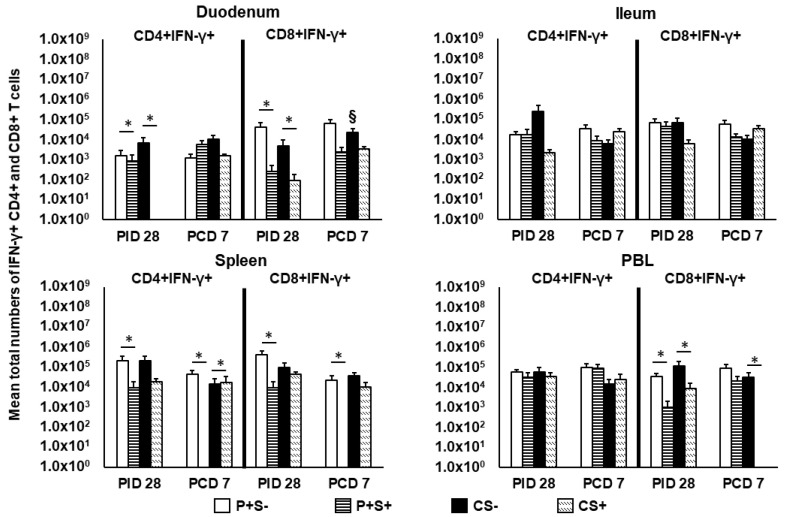
Total numbers of IFN-γ+ expressing CD4+ and CD8+ T cells in Gn pigs vaccinated with P particles with or without simvastatin feeding pre- and postchallenge. MNCs were gated as previously described [16] following in vitro NoV antigen stimulation and analyzed by flow cytometry (Supplementary Figure S4). Total numbers of IFN-γ producing CD4+ and CD8+ T cells following subtraction of isotype control and mock-stimulated background numbers are calculated. Data presented are total numbers plus standard errors of the means (n = 6 to 10) prechallenge and postchallenge in intestinal (duodenum, ileum) and systemic (spleen, PBL) tissues. See Figure 2 legend for statistical analysis and an explanation of the symbols indicating statistical significance.

We previously showed that NoV challenge induced a 37-fold expansion in the total numbers of duodenal CD4+IFN-γ+ T cells in previously NoV-infected pigs [16]. The results indicate the importance of an intestinal Th1 response in NoV immunity. In the present study, we identified IFN-γ+ producing CD4+ and CD8+ T cells in intestinal and systemic lymphoid tissues at PID 28 and PCD 7 by flow cytometry following in vitro stimulation with P particles. IFN-γ+ T cell numbers were determined after subtraction of isotype controls and mock-stimulated MNCs and are presented as mean total numbers (Figure 7). Pre-challenge at PID 28, simvastatin significantly reduced IFN-γ producing CD4+ and CD8+ T cell numbers in duodenum and CD8+IFN-γ+ T cell numbers in PBL compared to non-simvastatin pigs in both vaccinated and control pigs. In vaccinated pigs, simvastatin also significantly reduced IFN-γ producing CD4+ and CD8+ T cells in spleen. 

Post-challenge at PCD 7, P+S+ pigs had significantly lower numbers of CD4+IFN-γ+ and CD8+IFN-γ+ T cells in spleen compared to P+S− pigs. In fact, P+S+ pigs had no detectable CD4+IFN-γ+ and CD8+IFN-γ+ T cells in spleen post-challenge. Similarly, CS+ pigs had a complete lack of CD8+IFN-γ+ T cells in PBL, which were significantly lower than CS− pigs. Interestingly, CS+ pigs had significantly higher CD4+IFN-γ+ T cells in spleen compared to CS− pigs (Figure 7). Frequencies of the CD4+IFN-γ+ and CD8+IFN-γ+ T cells did not show any statistically significant difference among any treatment groups (data not shown). 

### 2.8. Simvastatin-Fed Pigs Had Reduced CD25−FoxP3+ and CD25+FoxP3+ Tregs in Duodenum

**Figure 8:**
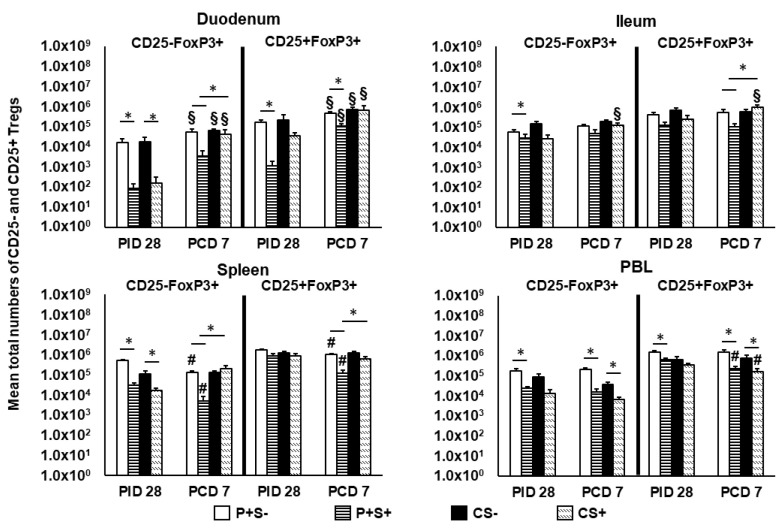
Total numbers of CD25− and CD25+ Tregs in Gn pigs vaccinated with P particles with or without simvastatin feeding pre- and post-challenge. Tregs were gated as previously described [16] following staining freshly on the day of cell isolation (Supplementary Figure S3). Total numbers of FoxP3+CD25− and FoxP3+CD25+ Tregs plus standard errors of the means (n = 6 to 10) among total MNCs prechallenge and postchallenge in intestinal (duodenum, ileum) and systemic (spleen, PBL) tissues are presented. See Figure 2 legend for statistical analysis and an explanation of the symbols indicating statistical significance. A number sign # indicates that the cell numbers decreased significantly following challenge in the same group (*p* < 0.05 by Kruskal-Wallis rank-sum test).

**Figure 9:**
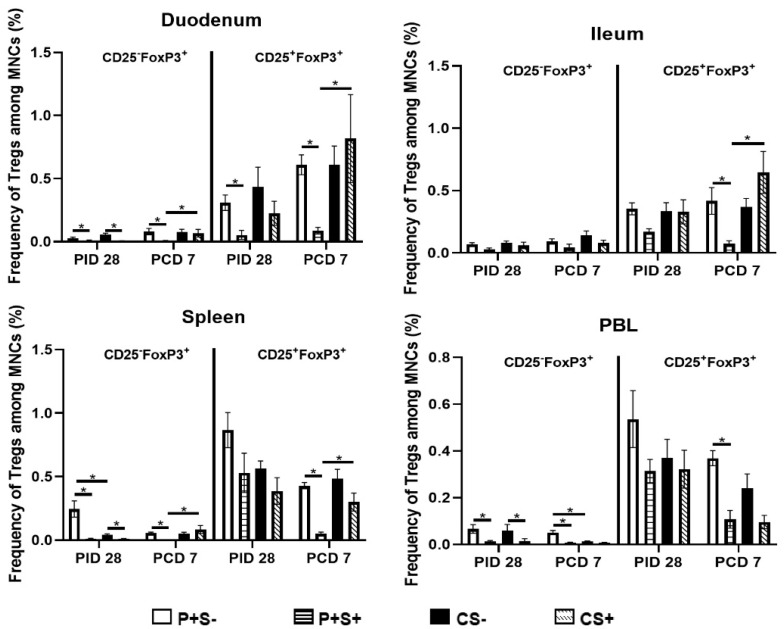
Frequencies of CD25^−^ or CD25^+^ Tregs in Gn pigs vaccinated with P particles with or without simvastatin feeding pre- and post-challenge. MNCs were stained freshly and gated as previously described [16] (Supplementary Figure S3). Data presented are mean frequencies +/− SEM (n = 6 to 10) of CD25^−^ or CD25^+^ cells expressing FoxP3^+^ among total MNCs in intestinal (duodenum, ileum) and systemic (spleen, PBL) tissue at PID 28 or PCD 7. An asterisk indicates statistical significance among groups for the same cell type at the same time point (*p* ≤ 0.05, Kruskal-Wallis rank-sum test).

Previously, we showed the importance of reducing Tregs in the duodenum in NoV protective immunity [16], though simvastatin has been shown to increase FoxP3 expression in murine CD4 T cells in vitro and in tumor cells in mice [43,44]. In the present study, we evaluated simvastatin’s effects on P particle-vaccine induced Tregs pre- and post-challenge. The mean total numbers and frequencies of CD25−FoxP3+ and CD25+FoxP3+ Tregs are shown in Figure 8 and Figure 9.

At PID 28, simvastatin feeding significantly reduced the numbers of CD25−FoxP3+ Tregs in duodenum and spleen of both vaccinated and control pigs. P+S+ pigs had significantly lower numbers of CD25−FoxP3+ Tregs in ileum and PBL and CD25+FoxP3+ Tregs in duodenum and PBL compared to P+S− pigs. Following challenge, simvastatin feeding significantly reduced the numbers of Tregs in PBL compared to non-simvastatin fed pigs. P+S+ pigs had significantly lower numbers of both Treg subsets in duodenum and spleen compared to P+S− pigs; P+S+ pigs also had significantly lower numbers of CD25+FoxP3+ Tregs in ileum compared to P+S− pigs. However, P+S+ pigs still had significantly reduced numbers of CD25−FoxP3+ Tregs in duodenum and spleen and CD25+FoxP3+ Tregs in ileum and spleen compared to CS+ pigs. CS+ pigs had significant increases in both Treg subsets in ileum compared to pre-challenge. Interestingly, all groups had increases or significant increases in CD25−FoxP3+ and CD25+FoxP3+ Tregs in duodenum following challenge. In spleen, both P+S+ and P+S− groups had significant decreases in both Treg subsets following challenge compared to pre-challenge numbers. In PBL, simvastatin-fed pigs (P+S+ and CS+) had significant decreases in CD25+FoxP3+ Tregs compared to pre-challenge.

When comparing frequencies of CD25− and CD25+ Tregs among P particle-vaccinated or control pigs with or without simvastatin pre- and post-challenge (Figure 9), the overall trends and statistical significances are very similar to comparing the numbers of CD25− and CD25+ Tregs in all tissues, except that in the duodenum the frequencies, not numbers, of CD25+FoxP3+ Treg of CS+ pigs were significantly higher than the CS− pigs at PCD 7 (Figure 8 and Figure 9). 

## 3. Discussion

In this study, we identified simvastatin’s effects on NoV vaccine-induced protection and T cell immune responses. First, we found that simvastatin impaired the P particle vaccine-induced protection against NoV shedding and abolished the protection against NoV diarrhea when compared to non-simvastatin treated Gn pigs in our previous study [16]. Secondly, we demonstrated that simvastatin impaired the P particle vaccine-induced T cell responses, especially those at the effector site of the gut-associated lymphoid tissues (duodenum). We showed that simvastatin impaired total MNC development in vivo and proliferating T cells in vitro, suggesting simvastatin can affect vaccine-induced immunity against NoV. 

Despite recent efforts, NoV T cell immunity remains understudied relative to B cell and antibody immunity [46]. Murine models have indicated that both CD4 and CD8 T cells are required for protection from murine norovirus (MNV) infection [47], CD8 T cells are required for MNV clearance [48] and CD4 T cells are a correlate of protection from MNV [49]. Previous studies have also indicated human NoVs induce strong Th1 response in humans [50] and Gn pigs [16]. The present study builds upon our previous findings in the role of T cells in NoV immunity. Among all the treatment groups, P particles without simvastatin (P+S−) had significantly higher numbers of Th cells, CTLs, activated CD4+ and CD8 T cells and IFN-γ producing CD4+ and CD8+ T cells in duodenum compared to P particle-vaccinated simvastatin-fed pigs (P+S+) at challenge (PID 28); additionally, it was the only group to have partial protection against NoV diarrhea [16]. This association between T cell responses and protection is similar to the findings in mice [48,49]. 

One of the goals of this study was to determine how simvastatin impacts the partial cross-variant protection (46.7% protection rate) against NoV diarrhea conferred by the P particle vaccine [16]. Simvastatin feeding totally abolished the previously observed protection as all the pigs in the P particles with simvastatin (P+S+) group had diarrhea. Simvastatin also resulted in significantly higher AUC of diarrhea compared to non-simvastatin fed pigs (8.8 vs. 5.4), eliminating the slight reduction in AUC of diarrhea among vaccinated pigs. It is important to note that although simvastatin alone can cause diarrhea in Gn pigs [41], the non-vaccinated control pigs with or without simvastatin did not differ significantly in the percent incidence (83%, 83%), duration (2.5, 1.8) or AUC (8.8, 6.7) of diarrhea. In the absence of simvastatin, the GII.4/2006b variant we used to challenge the pigs has been shown to be highly infectious [16,42]. The P particle vaccine did not reduce the incidence of virus shedding in either non-simvastatin (83%) or simvastatin (83%) fed pigs; however, P+S+ pigs still had significantly lowered numbers of days with virus shedding (1.7 vs. 4.2) and slightly decreased AUC of shedding (−2.3-fold) compared to control pigs. Hence, even with the presence of simvastatin, the P particle vaccine can still shorten the duration of virus shedding. Simvastatin feeding did not increase fecal virus shedding titers, which is consistent with the previous report in Gn pigs [41]. We noted a peculiar association between increased severities of diarrhea and decreased viral shedding titers. Increased watery diarrhea among simvastatin-fed pigs might have diluted the viruses or affected the quality of the fecal material collected by rectal swabs, leading to an underestimation of the amount of NoV shed. 

Another goal of this study was to identify the immune modulatory effects of simvastatin on P particle-induced T cell immunity. To better illustrate the impacts of simvastatin on T cell subsets, we presented both total number and frequency data of flow cytometry. When comparing the frequencies of the T cell subsets among different treatment groups, the trends are mostly same as comparing the total numbers. However, there are some statistically significant differences observed only in either the number or the frequency data. For example, frequencies of the CD4+IFN-γ+ and CD8+IFN-γ+ T cells did not show any statistically significant difference among any treatment groups, whereas the total numbers are significantly different in duodenum, spleen and blood between the simvastatin-fed and non-simvastatin-fed groups. This discrepancy emphasizes the necessity to examine both total number and frequency data of flow cytometry in immunophenotypic analysis when testing an immune modulating agent. 

Simvastatin significantly reduced numbers of all analyzed cell types in duodenum of vaccinated pigs at PID 28. Simvastatin further reduced the numbers of effector/memory CD4+IFN-γ+ and CD8+IFN-γ+ T cells in duodenum and effector/memory CD8+IFN-γ+ T cells in PBL at PID 28 compared to non-simvastatin fed pigs. Since simvastatin does not affect constitutive expression of MHC II [27], P particles were likely able to stimulate the initial innate and primary adaptive immune responses, but not memory immune responses. The latter is evident in the lack of Th and CTL responses in the spleen (Figure 3 and Figure 4) and the complete lack of splenic virus-specific IFN-γ+ producing T cells in P+S+ pigs following challenge (Figure 7), which indicates a lack of NoV-specific memory T cell response. 

The most important effects of simvastatin were determined to be on the development of total MNCs and proliferating T cells. Simvastatin reduced the total numbers of isolated MNCs in duodenum from vaccinated pigs pre-challenge and in PBL of both vaccinated and control pigs postchallenge, which likely contribute to the significantly lower numbers of all T cell types analyzed in duodenum at PID 28. Similarly, simvastatin-fed pigs had lower or significantly lower frequencies and numbers of Tregs in PBL at PCD 7. Simvastatin appears to impair the development of all MNCs, presumably including impaired antigen-presenting cells, T and B cells. 

Serum HBGA blocking antibodies are believed to be correlates of protection [49,51], but innate immunity has also been shown to be critical for control of MNV infection [52]. NoV immunity requires intact innate and adaptive immune responses; simvastatin’s impairment of total MNC development and immune activation is likely responsible for the abolishment of vaccine-induced partial protection. Further, simvastatin decreased the frequencies of non-specific proliferating T cells in blood and CD8+ T cells in duodenum. Simvastatin’s effects on NoV infection-induced and vaccine-induced B cell and innate immunity need to be investigation in future studies.

There are several limitations in this study. First, the immaturity of immune systems of neonatal Gn pigs may limit the direct extrapolation of the findings to elderly human populations. Second, we focused on studying T cell responses and did not measure the impact of simvastatin on antibody responses which are known to play important role in the immunity against NoV. More preclinical studies in animals and human clinical trials will need to be conducted to thoroughly determine how simvastatin impacts NoV pathogenesis and impairs vaccine immunogenicity and protective efficacy. These questions all have important practical implications. The impacts of concurrent treatment with simvastatin during NoV infection on the pathogenesis and the impact of simvastatin on humoral immune responses will be studied using the Gn pig model of NoV infection and diarrhea in the future.

In conclusion, simvastatin erased the partial protection conferred by the P particle vaccine and inhibited T cell development in the duodenum of vaccinated pigs. A robust immune response including T cells and B cells are required for viral clearance [47,48]. Since the elderly and aging are one of the primary target populations for a NoV vaccine [45] and the primary consumers of simvastatin type drugs, the implications of our findings that simvastatin can increase NoV-induced diarrhea and decrease the development of overall T cell responses are important in the development of preventive and therapeutic strategies against NoV gastroenteritis and maybe for other pathogens [53]. Simvastatin likely affects immune responses against many pathogens; the benefits and side effects of its use should be carefully analyzed and balanced. 

## 4. Materials and Methods

### 4.1. Virus

A pool of human stool containing GII.4/2006b variant 092895 (GenBank accession number KC990829) was collected at Cincinnati Children’s Hospital Medical Center by Dr. Xi Jiang’s laboratory. The stool pool was collected from a family with confirmed NoV gastroenteritis in 2008. The inoculum was processed by high-speed centrifugation as we previously described [42]. The ID_50_ of the inoculum for pigs at 33–34 days of age was 6.43 × 10^4^ viral RNA copies determined in our previous study [42] and 10 ID_50_ (6.43 × 10^5^) was used for challenging pigs with or without simvastatin treatment.

### 4.2. Vaccine

P particles from NoV GII.4 VA387 (a 1997 Farmington Hills variant) were prepared as previously described [54] and UV sterilized as previously described [16]. Synthetic MPL (Avanti Polar Lipids, Inc., Alabaster, AL, USA) and chitosan (Novamatrix, Sandvika, Norway) were diluted and filter sterilized as previously described [16]. Vaccines consisted of 100 µg P particles with 5 mg chitosan and 50 µg MPL adjuvants in TNC buffer [55] at a final volume of 1 ml. Sterility of all vaccine components was monitored as previously described [16].

### 4.3. Simvastatin Preparation

Simvastatin (Dr. Reddy’s Laboratories, Ltd, Hyderabad, Telangana, India) was prepared as previously described [42]. Tablets (80 mg) of simvastatin were dissolved in 100% ethanol for a final concentration of 8 mg/mL and filter sterilized. Serum cholesterol concentrations in Gn pigs pre- and post-feeding were evaluated by the Virginia-Maryland College of Veterinary Medicine hospital laboratory to verify simvastatin’s effects. Data were presented as the mean serum cholesterol levels in mg/dL.

### 4.4. Simvastatin Treatment, Vaccination and Virus Inoculation of Gn Pigs

Near-term Large White cross pigs from the same sources were derived via hysterectomy and maintained in germ-free isolator units as previously described [56]. Pigs (both male and female) were randomly assigned into four groups: (1) P particles without simvastatin (P+S−), (2) P particles with simvastatin (P+S+), (3) Control without simvastatin (CS−) and (4) Control with simvastatin (CS+). Each group was composed of 6 to 10 pigs; simvastatin groups were conducted in sequential experiments after the non-simvastatin fed groups (which were previously reported [16]) and were vaccinated and challenged with the same vaccine and virus preparations, fed the same diet and kept under the same housing conditions in the same facility. The challenge virus inoculums were prepared from the single clinical isolate to avoid variabilities. All pigs in the P+ groups were intranasally inoculated with 3 doses of the vaccine using mucosal atomization devices (MADs, LMA North America, Inc. San Diego, CA, USA) at post-partum day (PPD) 5 (post-inoculation day [PID] 0), PID 10 and PID 21. Control pigs received adjuvants alone at the same time points. Simvastatin-treated pigs were orally inoculated with 8 mg/day/pig of simvastatin in a diluent for 11 days before challenge (PID 17–27, approximately 1.5–2.0 kg of body weight). At PID 28, a subset of pigs from each group was challenged with 10 ID_50_ of the GII.4/2006b variant 092895 at PID 28 (post-challenge day [PCD] 0). Four ml of 200 mM sodium bicarbonate were given to pigs 10 minutes before NoV oral inoculation to reduce gastric acidity. Challenged pigs were monitored daily for diarrhea and virus shedding until PCD 7. All pigs were euthanized at PID 28 (pre-challenge) or PCD 7 (post-challenge). Mononuclear cells (MNCs) from duodenum (20 cm in length), ileum (20 cm in length), spleen (whole organ) and blood (70 ml) were isolated as previously described [57]. The total numbers of MNCs were calculated by multiplying the volume and concentration of cells isolated from each tissue. 

### 4.5. Assessment of NoV Shedding and Diarrhea

Rectal swabs were collected daily from PCD 1 to 7. Diarrhea scores were given based on our previously used scaling system: 0, normal; 1, pasty; 2, semi-liquid; 3, liquid. Scores of ≥2 were considered diarrheic [42]. Pigs that had a score of 2 or greater for at least one day from PCD 1 to PCD 7 were considered positive for diarrhea. Virus shedding was detected using TaqMan® real-time RT-PCR as previously described [42]. Pigs were considered positive for shedding if NoV was detected in a stool sample for at least one day from PCD 1 to PCD 7. 

Diarrhea severity and shedding are presented as areas under the curve (AUCs). AUCs were calculated from the line graphs using the daily diarrhea score or daily shedding titer (Y-axis) and the days of diarrhea or virus shedding (X-axis). AUCs were calculated for disease and shedding from PCD 1 through PCD 7 using GraphPad Prism 6.

### 4.6. In Vitro MNC Proliferation Assay

Flow cytometry was used to determine how simvastatin affects CD4+ and CD8+ T cell proliferation in intestinal (duodenum, ileum) and systemic (spleen) tissues and peripheral blood lymphocytes (PBL) of Gn pigs. MNCs were isolated from non-simvastatin fed, P particle-vaccinated and NoV challenged pigs at PCD 7. MNCs were cultured with or without simvastatin (1 µM; concentration determined in pilot study) for 5 days in E-RPMI media in 5% CO2 and 37 °C. MNCs were stimulated with P particles or PHA to measure virus-specific T cell and total T cell proliferation, respectively. Bromodeoxyuridine (BrdU, BD Biosciences, San Jose, CA, USA) was added for the final 24 h of culture. MNCs were harvested and stained for CD3, CD4 and CD8 and PE anti-BrdU (BD Pharmingen, San Diego, CA, USA). MNCs stained with isotype-matched irrelevant antibodies were used to establish positive and negative gates. Data are presented as mean frequencies among CD3+ T cells. At least 100,000 events were acquired using a BD FACSAria flow cytometer (BD Biosciences) and data were analyzed using FlowJo 7.6.4 software (Tree Star, Inc. Ashland, OR, USA). 

### 4.7. Flow Cytometry Analysis of Total Th, CTL and IFN-γ Producing CD4+ and CD8+ T Cells

Flow cytometry was used to determine the total numbers and frequencies of CD4+ and CD8+ T cells and IFN-γ producing CD4+ and CD8+ T cells in the intestinal (duodenum and ileum) and systemic (spleen) tissues and peripheral blood lymphocytes (PBL) of Gn pigs as previously described [16]. Th and CTLs were defined as CD3+CD4+ and CD3+CD8+, respectively. Cells were stimulated with P particles in vitro for 17 h and stained as previously described [16,58,59]. Total numbers of each T cell subset per tissue were calculated as previously described [16]. Data are presented as total numbers and frequencies of the T cells from each tissue. Numbers or frequency of IFN-γ producing T cells are presented as mean adjusted numbers or frequency following removal of mock-stimulated MNCs and isotype-matched irrelevant controls. At least 100,000 events were collected using a BD FACSAria flow cytometer (BD Biosciences) and analyzed using FlowJo 7.6.4 software (Tree Star, Inc.).

### 4.8. Flow Cytometry Analysis of Activated Non-Regulatory (FoxP3−) and IL-10 and TGF-β Producing Tregs (FoxP3+) Cells

MNCs were stained freshly on the day of isolation for activated non-regulatory T cells and Tregs as previously described [58]. Activated non-regulatory T cells were defined as CD25+FoxP3− T cells and Tregs were defined as CD25-FoxP3+ and CD25+FoxP3+ T cells as previously described [16]. Total numbers of Tregs and IL-10 and TGF-β producing Tregs per tissue were calculated as previously described [16]. Isotype-matched irrelevant antibodies were used to establish positive and negative gates for FoxP3. At least 100,000 events were collected using a BD FACSAria flow cytometer (BD Biosciences) and analyzed using FlowJo 7.6.4 software (Tree Star, Inc.).

### 4.9. Statistical Analysis 

One-way analysis of variance (ANOVA)-general linear model (GLM) followed by Duncan’s multiple range test was used to compare mean durations of diarrhea and shedding. Kruskal–Wallis rank-sum test was used to compare diarrhea and viral shedding AUCs and numbers of T cell subsets. A two-tailed paired Student’s *t*-test was used to compare mean serum cholesterol levels following simvastatin feeding. All statistical significance was assessed at *p* < 0.05. All statistical analyses were performed using SAS Program 9.3 (SAS Institute, Cary, NC, USA).

## Figures and Tables

**Table 1:** Clinical signs and protective efficacy in P particle-vaccinated Gn pigs after challenge with GII.4 2006b NoV ^a^.

		Diarrhea ^b^				Virus Shedding			
Group *	n	Percent of pigs with diarrhea (no. of pigs with diarrhea/total no.)	Mean no. of days with diarrhea ^c^ (SEM)	Mean AUC (SEM) **	Fold reduction in AUC	Percent of pigs shed virus (no. of pigs with shedding/total no.)	Mean no. of days with shedding ^c^ (SEM) **	Mean AUC	Fold reduction in AUC
P+S+	6	100% (6/6)	3.0 (0.7)	8.8 (1.1) ^A^	0.0	83% (5/6)	1.7 (0.4) ^B^	1.87 × 10^4^	−2.3
CS+	6	83% (5/6)	2.5 (0.6)	8.8 (0.7) ^A^	NA	83% (5/6)	4.2 (1.3) ^A^	4.22 × 10^4^	NA

^a^ Gn pigs were challenged with 10 ID_50_ of a human NoV GII.4 2006b variant 092895 at 33–34 days of age (PID 28). Rectal swabs were collected daily after challenge to determine diarrhea and virus shedding by real-time RT-qPCR. Virus shedding was also detected in intestinal contents. ^b^ Fecal scoring system: 0, solid; 1, pasty; 2, semi-liquid; 3, liquid. Pigs with scores of 2 or higher were considered diarrheic. ^c^ From postchallenge day (PCD) 1 to PCD 7. NA, no applicable. * Abbreviated group names: P+S+, simvastatin-fed P particle-vaccinated; CS+, simvastatin-fed placebo control. Pigs in all groups received MPL/chitosan adjuvants. ** Means in the same column followed by different letters (^A, B^) differ significantly (One way ANOVA, *p* < 0.05); while shared letters indicate no significant difference.

## Data Availability

All data supporting reported results can be found in this manuscript.

## Supplementary Materials

The following are available online at https://www.mdpi.com/article/10.3390/pathogens10070829/s1, Figure S1: Gating and staining strategy for CD3+CD4+BrdU+ and CD3+CD8+ BrdU+ T cells; Figure S2: Gating strategy and representing dot plots of Th and CTL in different tissues; Figure S3: Gating strategy and representing dot plots of non-regulatory (FoxP3-), activated CD4+CD25+ and CD8+CD25+ T cells and CD25+/CD25-CD4+FoxP3+ Treg cells in different tissues; Figure S4: Gating strategy and representing dot plots of IFN-γ+ cells among CD3+CD4+ and CD3+CD8+ cells in different tissues.
