# Supplementary material for: Simvastatin Reduces Protection and Intestinal T Cell Responses Induced by a Norovirus P Particle Vaccine in Gnotobiotic Pigs
Source: Pathogens. 2021 Jul 1;10(7):829. doi: 10.3390/pathogens10070829 (PMC8308729; doi:10.3390/pathogens10070829)

Supplementary Figure S1. Gating and staining strategy for CD3+CD4+BrdU+ and CD3+CD8+ BrdU+ T cells

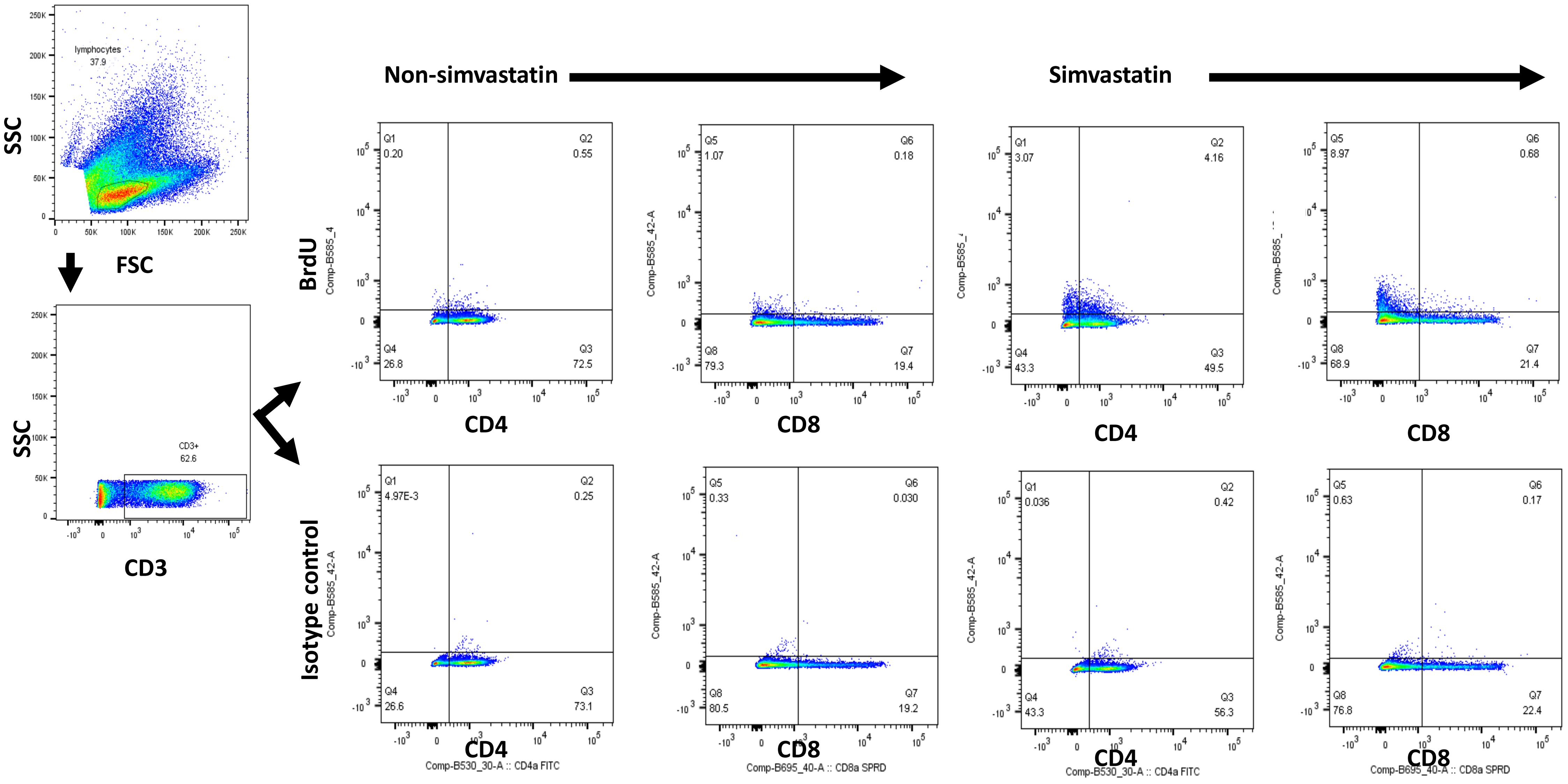

Supplementary Figure S2. Gating strategy and representing dot plots of Th and CTL in different tissues.

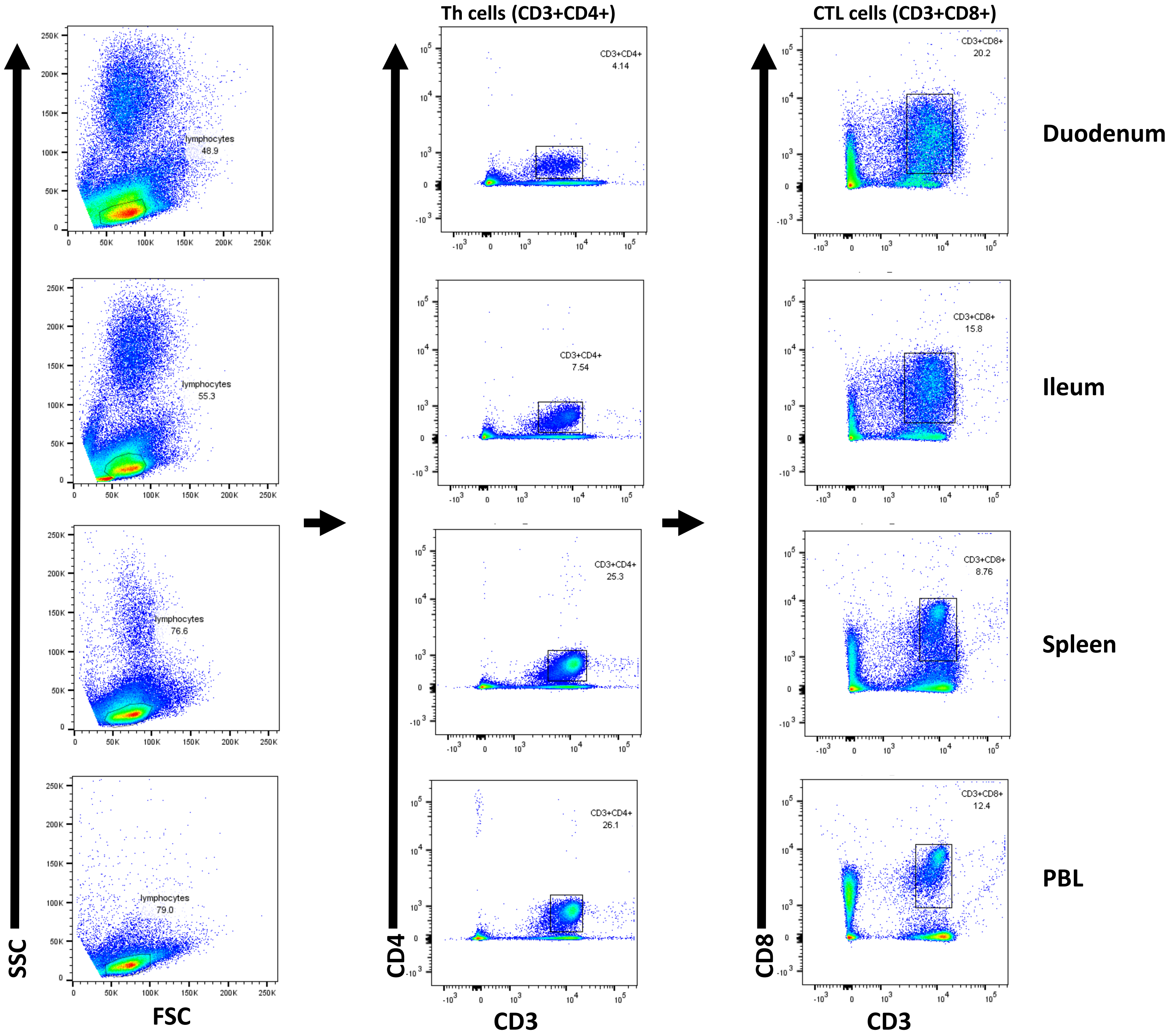

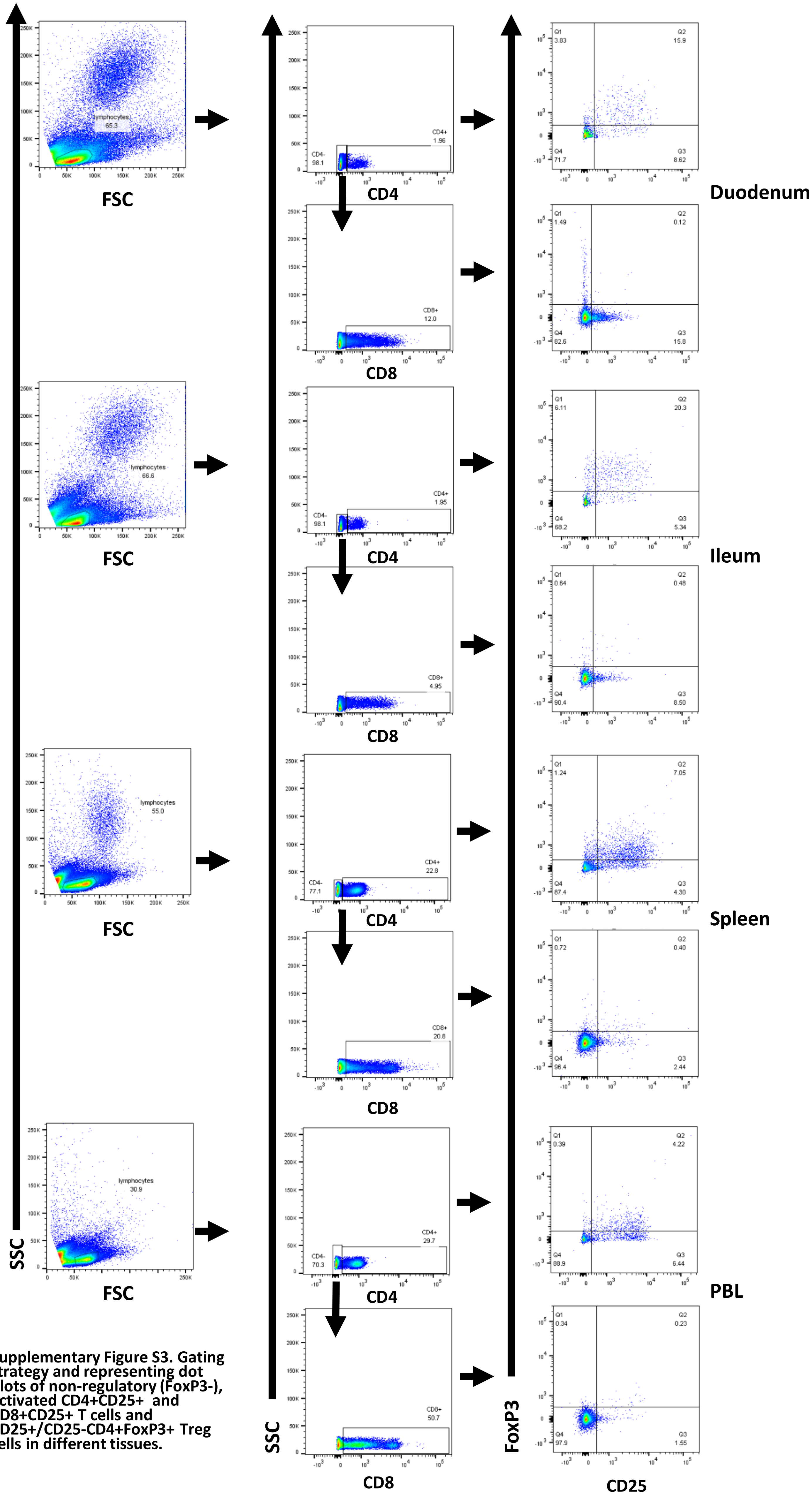

Supplementary Figure S3. Gating strategy and representing dot plots of non-regulatory (FoxP3-), activated CD4+CD25+ and CD8+CD25+ T cells and CD25+/CD25-CD4+FoxP3+ Treg cells in different tissues.

Supplementary Figure S4 Gating strategy and representing dot plots of IFN-γ+ cells among CD3+CD4+ and CD3+CD8+ cells in different tissues.

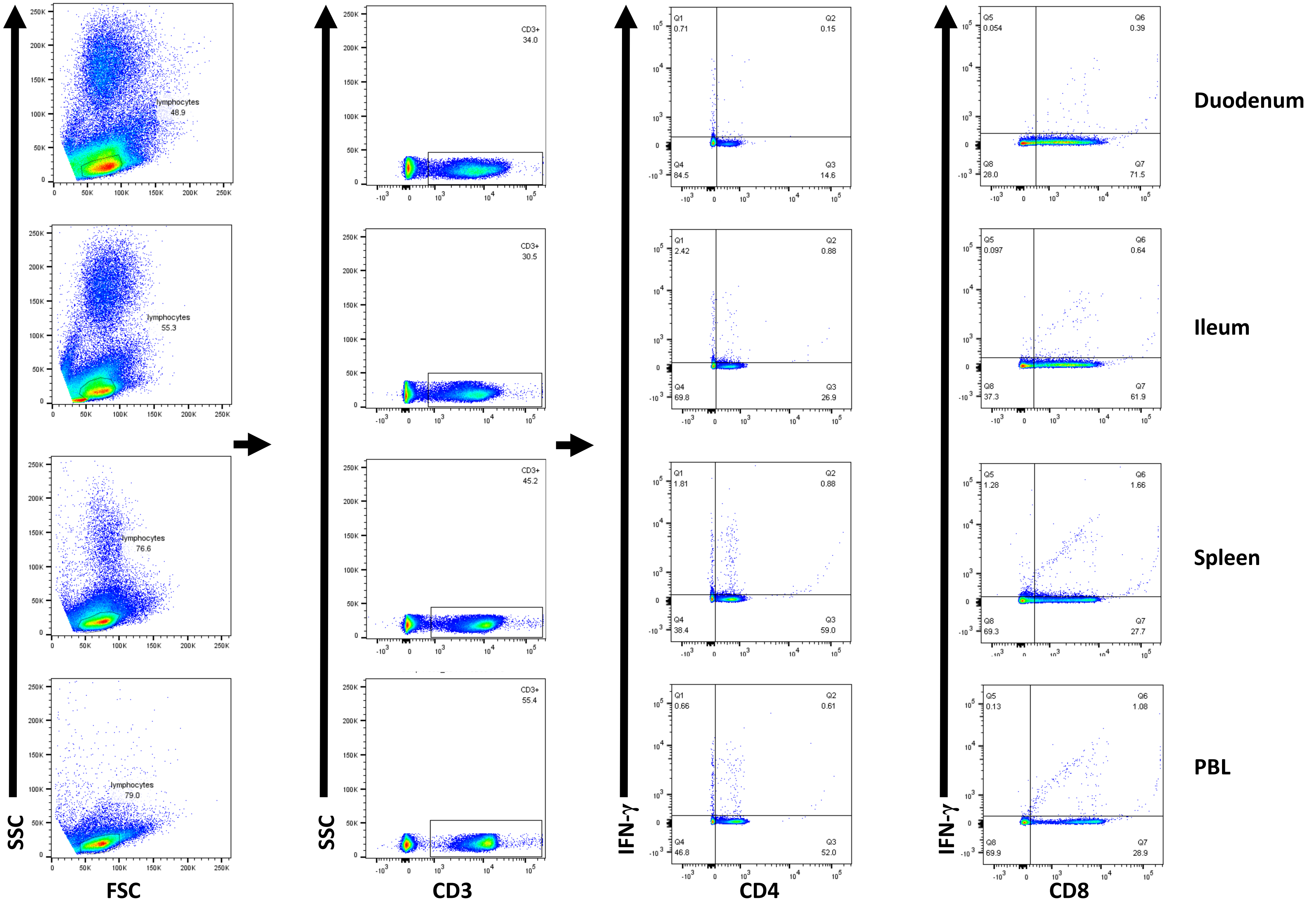

Supplement: Supplementary file 1 [file pathogens-10-00829-s001.zip › pathogens-1236308-supplementary.pdf]
